# Synthesis, in vitro urease inhibitory potential and molecular docking study of benzofuran-based-thiazoldinone analogues

**DOI:** 10.1038/s41598-020-67414-7

**Published:** 2020-06-30

**Authors:** Muhammad Taha, Fazal Rahim, Hayat Ullah, Abdul Wadood, Rai Khalid Farooq, Syed Adnan Ali Shah, Muhammad Nawaz, Zainul Amiruddin Zakaria

**Affiliations:** 1 Department of Clinical Pharmacy, Institute for Research and Medical Consultations (IRMC), Imam Abdulrahman Bin Faisal University, P.O. Box 1982, Dammam, 31441 Saudi Arabia; 2 Department of Chemistry, Hazara University, Mansehra, Khyber Pakhtunkhwa 21300 Pakistan; 3 Department of Biochemistry, Abdul Wali Khan University Mardan, Mardan, 23200 Pakistan; 4 Department of Neuroscience Research, Institute for Research and Medical Consultations (IRMC), Imam Abdulrahman Bin Faisal University, P.O. Box 1982, Dammam, 31441 Saudi Arabia; 5 Atta-ur-Rahman Institute for Natural Product Discovery, Universiti Teknologi MARA, Cawangan Selangor Kampus Puncak Alam, 42300 Bandar Puncak Alam, Selangor D. E. Malaysia; 6 Faculty of Pharmacy, Universiti Teknologi MARA, Cawangan Selangor Kampus Puncak Alam, 42300 Bandar Puncak Alam, Selangor Darul Ehsan Malaysia; 7 Department of Nano-Medicine Research, Institute for Research and Medical Consultations (IRMC), Imam Abdulrahman Bin Faisal University, P.O. Box 1982, Dammam, 31441 Saudi Arabia; 8 Department of Biomedical Science, Faculty of Medicine and Health Sciences, Universiti Putra Malaysia, 43400 Serdang, Selangor Malaysia; 9 Halal Institute Research Institute, Universiti Putra Malaysia, 43400 Serdang, Selangor Malaysia

**Keywords:** Drug discovery, Chemistry

## Abstract

In continuation of our work on enzyme inhibition, the benzofuran-based-thiazoldinone analogues (**1–14**) were synthesized, characterized by HREI-MS, ^1^H and ^13^CNMR and evaluated for urease inhibition. Compounds **1–14** exhibited a varying degree of urease inhibitory activity with IC_50_ values between 1.2 ± 0.01 to 23.50 ± 0.70 µM when compared with standard drug thiourea having IC_50_ value 21.40 ± 0.21 µM. Compound **1, 3, 5** and **8** showed significant inhibitory effects with IC_50_ values 1.2 ± 0.01, 2.20 ± 0.01, 1.40 ± 0.01 and 2.90 ± 0.01 µM respectively, better than the rest of the series. A structure activity relationship (SAR) of this series has been established based on electronic effects and position of different substituents present on phenyl ring. Molecular docking studies were performed to understand the binding interaction of the compounds.

## Introduction

Benzofuran scaffolds due to its profound chemotherapeutic, physiological properties and their dynamic nature has attracted the attention of chemist during last few years^1^. Acting as versatile scaffolds, benzofuran derivatives can be used to synthesize potentially new therapeutic agents^2^. These scaffolds exhibited biological properties such as antimicrobial^3^, analgesic^4^, anti-hyperglycemic^5^, anti-parasitic^6^, antitumor and kinase inhibitors^7^. Besides these properties benzofuran scaffolds also found application as oxidant^8^, fluorescent sensor^9^, brightening agent, antioxidant and in other field of chemistry and agriculture^10^.

Urease is a metalloenzyme contain nickel that is responsible for catalyzing urea hydrolysis to ammonia and carbamate, the latter spontaneously hydrolyzing to carbonic acid and a second molecule of ammonia in an uncatalyzed reaction^11^. Ammonia molecules thus formed are protonated by water at physiological pH, whereas the carbonic acid dissociates and causes an increase in pH.
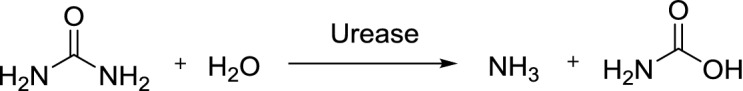



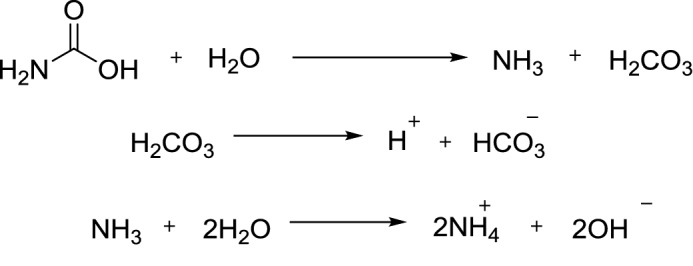


Urease enzyme is involved to function by using urea as nitrogen source^12,13^. Urease is responsible for the one of the major diseases induced by *Helicobacter pylori*, allowing them to survive inside the stomach at low pH thus play a vital role in peptic and gastric ulcer pathogenesis, apart from cancer too^12^. Urease play a key role in the infection stones formation that equally take part in pathogenesis of pylenephritis, hepatic encephalopathy, urolithiasis, urinary catheter encrustation and hepatic coma^14^. Certain secondary complication like ulcer, pus formation and infectious diseases can be treated by inhibiting urease enzyme with help of specific potent inhibitors^15^. Previously published urease inhibitors are 2-acylated and sulfonated 4-hydroxycoumarins, *bis*-indolylmethane thiosemicarbazides and benzimidazole analogues^16–18^.

**Figure 1:**
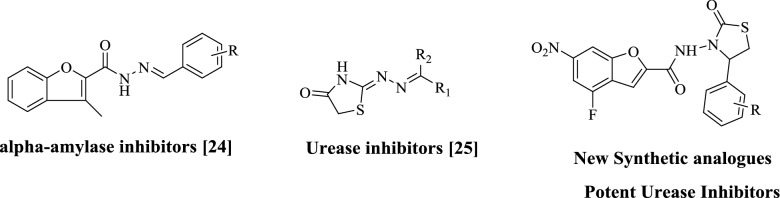
Rational of the current study.

Our research group already has been working on design and synthesis of heterocyclic compounds in search of lead compound since many years and we had some promising outcomes^19–30^. We had already reported benzofuran hydrazone scaffolds as novel *α*-amylase inhibitors^31^ (Fig. 1) and thiazolidinone scaffolds as potent urease inhibitors^32^ but still improvement needed to identify some potential lead compounds for more advance research in future. Keeping in view, here in this study we have design synthesis and biological screening of benzofuran bearing thiazolidinone scaffolds (**1–14**) as urease inhibitors.

## Results and discussion

### Chemistry

**Scheme 1:**
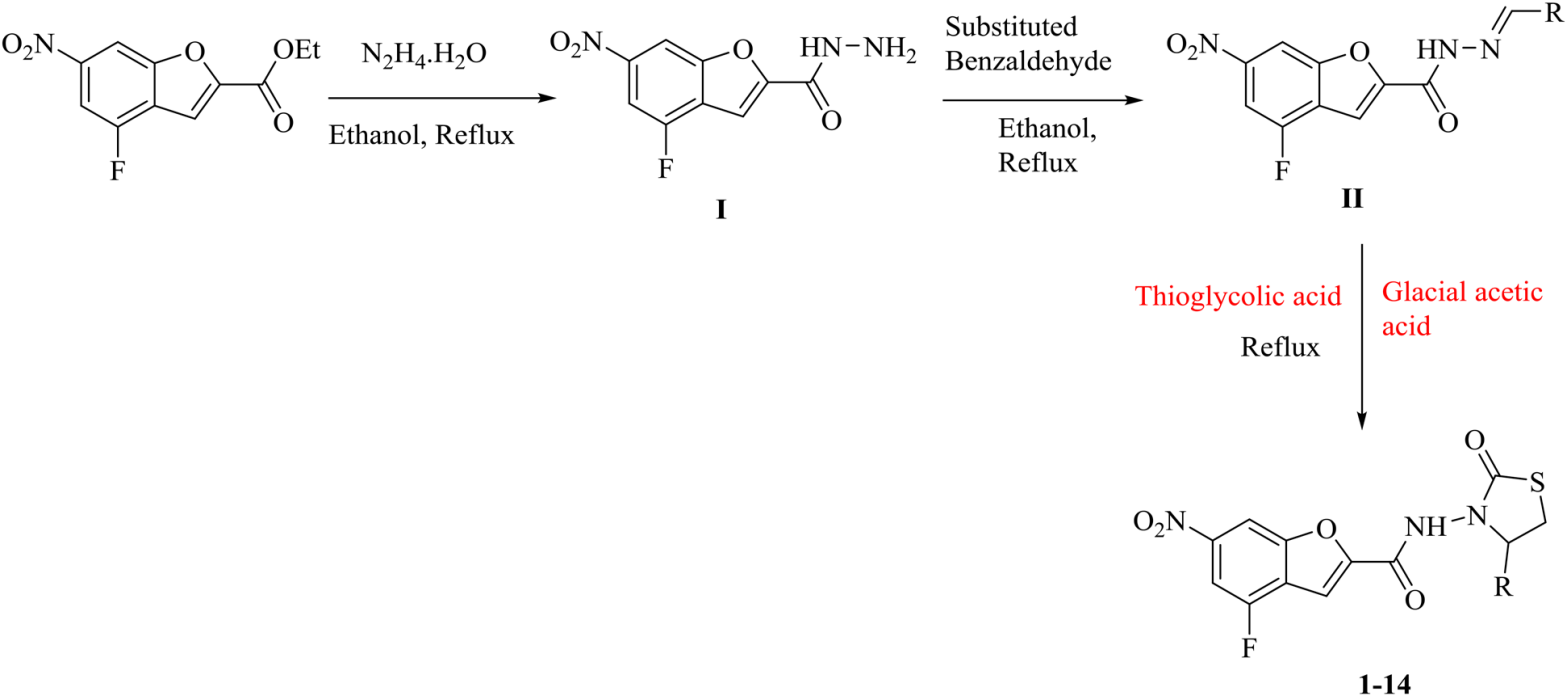
Synthesis of benzofuran bearing thiazoldinone analogues.

4-fluoro-6-nitrobenzofuran-2-carboxylate (1 mmol) was reacted and refluxed with hydrazine hydrate (20 ml) in EtOH (15 ml) to yield 4-fluoro-6-nitrobenzofuran-2-carbohydrazide as intermediated product (**I**). Then with different substituted benzaldehyde, this intermediate product (**I**) was reacted and refluxed in EtOH (15 ml) to yield 4-fluoro-6-nitrobenzofuran-2-carbohydrazide as 2nd intermediate product (**II**). In final step, thioglycolic acid in the presence of few drops of glacial acetic acid was reacted and refluxed with intermediate product (**II**) to yield desired products of benzofuran bearing thiazolidinone analogues (**1–14**) (Scheme 1).

### In vitro* urease inhibitory potential*

The synthesized benzofuran bearing thiazolidinone scaffolds (**1–14**) were evaluated against urease enzyme. Varied degree of potential was exhibited by all scaffolds ranging between 1.2 ± 0.01 to 23.50 ± 0.70 µM comparing with standard drug thiourea (IC_50_ = 21.40 ± 0.21 µM). Excellent inhibitory potential was exhibited by all synthesized scaffolds. Structure activity relationship (SAR) was established based on different substitution pattern on phenyl ring (Table 1).

**Table 1:**
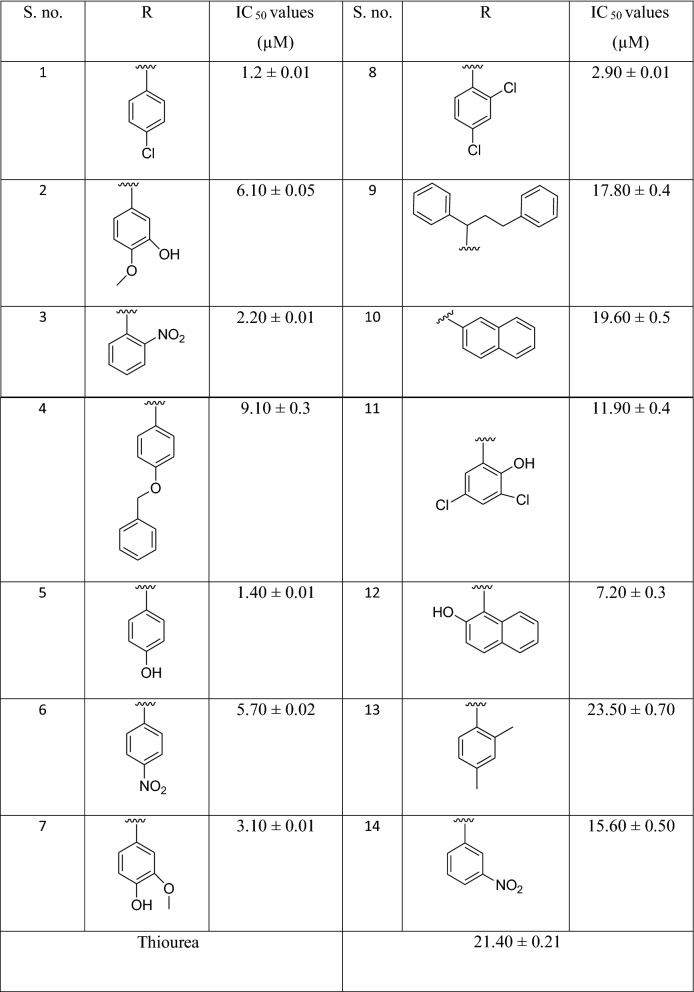
Urease activity and different ‘‘R’’ group of benzofuran bearing thiazoldinone analogues.

Scaffold **1** (IC_50_ = 1.2 ± 0.01 μM) that has 4-chloro moiety as substituent on phenyl ring was most potent scaffold among the whole series. An excellent potential of this scaffold might be due to the existence of 4-chloro moiety on phenyl ring which is electron withdrawing in nature.

If we compare scaffold **3** (IC_50_ = 2.2 ± 0.01 μM) that has NO_2_ moiety at positon-2 on phenyl ring with scaffold **6** (IC_50_ = 5.70 ± 0.02 μM) that has NO_2_ moiety at position-4 on phenyl ring and scaffold **14** (IC_50_ = 15.60 ± 0.50 μM) that has NO_2_ moiety at position-3 on phenyl ring. All these three NO_2_ substituted scaffolds showed that the potential difference may be due to the different positions of NO_2_ moiety.

If we compare scaffold **2** (IC_50_ = 6.10 ± 0.05 μM) that have hydroxyl at 3- position and methoxy moiety at 4-position on the phenyl ring with scaffold **7** (IC_50_ = 3.10 ± 0.01 μM) that have hydroxyl at 4-position and methoxy group at 3-position on the phenyl ring. The potential difference between these two scaffolds may be due to different positions of hydroxyl and methoxy moiety on the phenyl ring.

Overall it has been concluded that either electron withdrawing groups (EWG) or electron donating groups (EDG) on phenyl ring exhibited good potency but slightly difference in their potency was also mostly affected due to positions of substituents.

### Molecular docking

In catalytic pocket of urease enzyme to explore the binding modes of the synthesized scaffolds the Molecular Operating Environment (MOE) package^33^ was used to study molecular docking study. With the help of builder tool executed in MOE package, 3D structural coordinates of scaffolds were generated. By using default parameter of MOE, energy of the synthesized scaffolds was minimized, and all 3D coordinates of the scaffolds were protonated. Using PDB code 4UBP, from the online free server protein databank (www.rcsb.org), the crystallographic 3D structure of urease enzyme was retrieved. Next, the structure was added to MOE for protonation, energy to get the stable conformation of protein with the help of default parameter of MOE package. Finally, using the default parameters of MOE package to perform molecular docking studies i.e., Placement: Rescoring 1, Triangle Matcher, Refinement, London dG: Rescoring 2, Forcefield: GBV1/WSA. For each synthesized scaffold, total 10 conformations were allowed to be form. Later, for addition analysis the top ranked conformations were selected.

### Docking study

**Figure 2:**
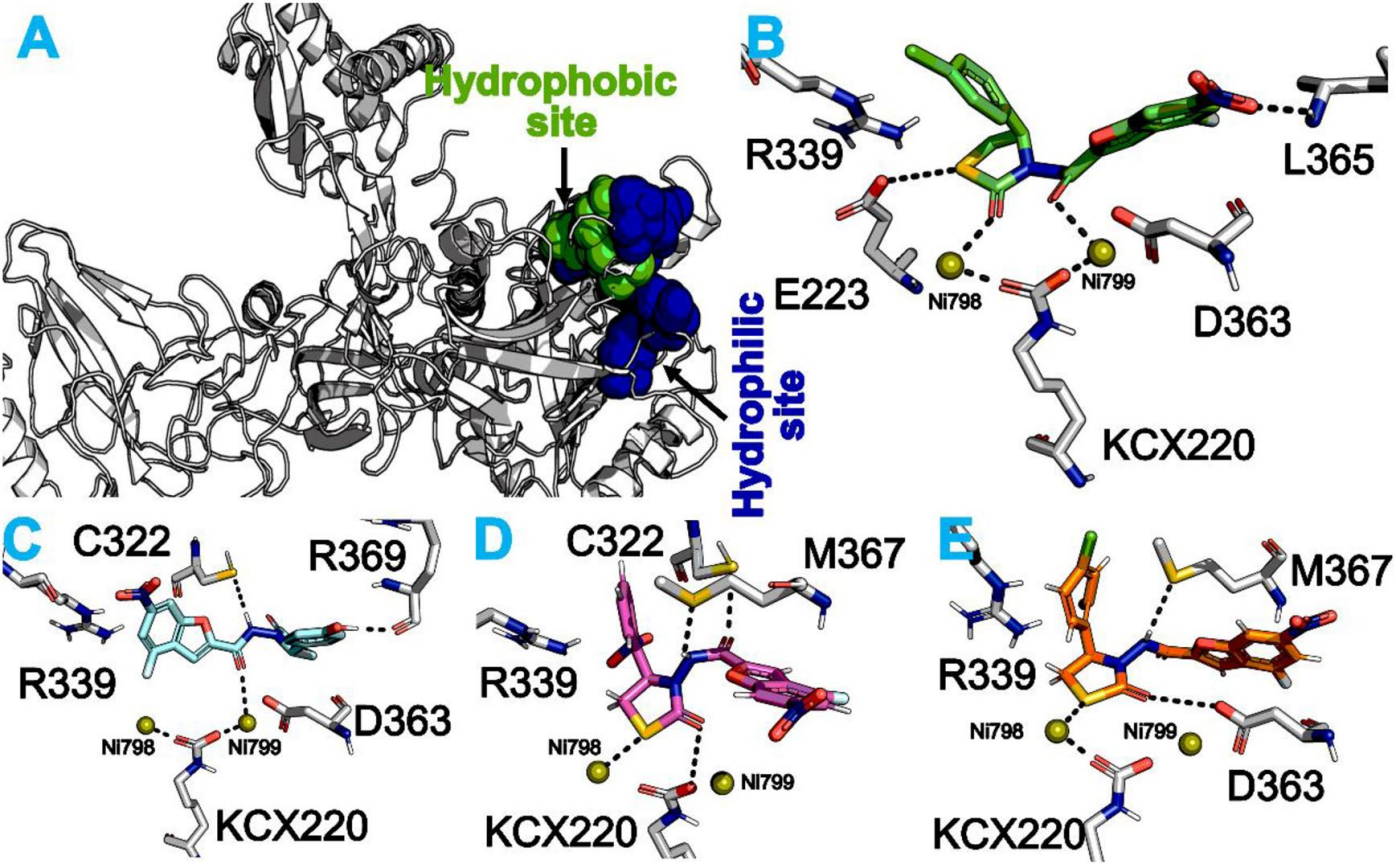
Urease enzyme (PDB code 4UBP) catalytic site binding mode analysis of the most potent scaffolds. (**A**) Urease enzyme (PDB ID 4UBP) surface representation, hydrophilic and hydrophobic regions are colored to blue and green, furthermore, two Nickle ions (799 and 798) are shown in sphere. In dark black color dotted lines, the hydrogen bonding is shown, and π-stacking interactions are shown by both sided arrows. (**B**) Scaffold-**1** mode of interaction (**C**) Scaffold-**5** and (**D**) Scaffold-**3** and (**E**) Scaffold-**8**.

In order to explain the synthesized scaffolds binding pattern, molecular docking studies was conducted in the urease enzyme catalytic pocket (PDB code 4UBP). The urease enzyme catalytic site contains both hydrophobic and hydrophilic site residues (Fig. 2A). The hydrophilic site includes 223, 494, 323, 324, 249, G166, D224, R339 and H315, while hydrophilic site composed of 366, A170, C322, L319 and K169. Furthermore, these two Nickle ions (Ni799 and Ni798) conjointly compete a key role by linking the ligands and key residues. Though, the results of docking showed a well fit sketch of binding in catalytic site and with catalytic residues adopt the most favorable interaction (Fig. 2). Usually, through the binding mode analysis it showed not only that the inhibitory potential of those scaffold was excellent that possess electron withdrawing groups (EWG’s) at *para* position, but also with catalytic residues showed favorable interactions. In contrast, among the series in our current study some scaffolds which possess -di- (EWG’s) at *para* and *ortho* position that exhibited less potency against urease enzyme. The reason for the decrease in enzyme activity might be that the halides at *para*, *ortho* directing groups most likely prefer to deactivate the benzene ring that result in potency decrease^34^. Furthermore, the scaffolds among the series that has electron donating groups (EDG’s) possess good inhibitory potential against urease enzyme. Though binding mode analysis of most potent scaffold **1** (1.2 ± 0.01 μM) showed the fit well pattern of binding in catalytic site, therefore the scaffold was unable to adopt favorable ionic and other interactions (i.e. hydrophobic, hydrogen bond etc.) with catalytic residues like `D363, E223, L365, R339 and with the modified residue KCX220. Furthermore, the two Ni ions (Ni799 & Ni798) accept ionic bond with O13 and S19 of the corresponding scaffold, and additionally improve the potential against urease enzyme (Fig. 2B). High potential of this scaffold might be due to the reason that the attached EWG-1 decreases the π-system electronic density thus making the π-system more electrophilic and hence initiate the partial positive charge on benzene, which make this benzene unable to adopt π-interaction with catalytic residues (R339).

In same manner, the second most potent scaffold **5** that has electron donating group (EDG-OH) among series through binding modes showed the similar binding pattern (Fig. 2C) upon comparison with most potent scaffold **1**. In both scaffolds (**1** and **5**) the difference perceived through binding modes analysis are not only ionic interaction of both Ni ions and modified residues KCX220 but also due to variation in EDG and EWG. Scaffold **5** showed ionic interaction only with single Ni ion while in case of scaffold **1** interaction was with both Ni ions as well as KCX220 residues.

In same manner, the scaffolds **3** and **8** through binding mode analysis showed similar binding mode (Fig. 2D, E). With experimental results the docking results are related well based on key residues with ligands multiple interactions of the urease enzyme. Docking poses of all active scaffolds were computatiionally reserved the catalytic potentials of urease by firmly binding through hydrogen bonding, strong hydrophobic and polar interactions with key residues.

## Conclusion

Benzofuran bearing thiazolidinone scaffolds (**1–14**) have synthesized in excellent to moderate yield (68–88%). These synthesized scaffolds were evaluated for urease inhibitory potential. All the synthesized scaffolds showed excellent to good inhibitory potential against urease inhibition with IC_50_ value ranging between 1.2 ± 0.01 to 23.50 ± 0.70 µM as compare to standard thiourea having IC_50_ value 21.40 ± 0.21 µM. SAR of potent scaffolds was established and were confirmed through molecular docking studies. Among the series, scaffold **1** was found potent urease inhibitor which can act as lead scaffold for further development of drug.

## Material and methods

### General method for the synthesis of benzofuran-2-carbohydrazide bearing thiazoldinones analogs

4-fluoro-6-nitrobenzofuran-2-carboxylate was reacted and refluxed with excess of hydrazine hydrate in EtOH to yield 4-fluoro-6-nitrobenzofuran-2-carbohydrazide as intermediated product (**I**). Then with different substituted benzaldehyde, this intermediate product (**I**) was reacted and refluxed in EtOH to yield 4-fluoro-6-nitrobenzofuran-2-carbohydrazide as 2nd intermediate product (**II**). In final step, thioglycolic acid in the presence of glacial acetic acid was reacted and refluxed with intermediate product (**II**) to yield desired products of benzofuran bearing thiazolidinone analogues (**1–14**).

#### N-(4-(4-chlorophenyl)-2-oxothiazolidin-3-yl)-4-fluoro-6-nitrobenzofuran-2-carboxamide (1)

Yield: 68%. ^1^HNMR (500 MHz, DMSO-*d*_6_): *δ* 10.05 (s, 1H, NH), 8.81 (s, 1H, Ar), 8.35 (d, *J* = 2.9 Hz, 1H, Ar), 7.94 (d, *J* = 5.15 Hz, 2H, Ar), 7.87 (s. 1H, Ar), 7.42 (t, *J* = 0.7, 1.35 Hz, 2H, Ar), 5.29 (s, 1H, CH), 3.75 (d, J = 1.8 Hz, 2H, CH_2_). ^13^CNMR (125 MHz, DMSO-*d*_*6*_): *δ* 165.2, 162.5, 158.5, 157.7, 152.1, 150.2, 141.6, 132.3, 128.6, 128.6, 127.2, 127.2, 124.3, 110.1, 107.4, 104.5, 64.5, 33.4. HREI-MS: m/z calcd for C_18_H_11_ClFN_3_O_5_S [M]^+^ 435.0092, Found 435.0078.

#### 4-Fluoro-N-(4-(3-hydroxy-4-methoxyphenyl)-2-oxothiazolidin-3-yl)-6-nitrobenzofuran-2-carboxamide (2)

Yield: 71%. 1HNMR (500 MHz, DMSO-*d*_6_): *δ* 10.06 (s, 1H, NH), 8.83 (s, 1H, Ar), 8.36 (t, *J* = 9.45 Hz, 1H, Ar), 7.96 (d, *J* = 7.45 Hz, 2H, Ar), 7.91 (s. 1H, Ar), 7.41 (t, *J* = 5.7, 10.05 Hz, 1H, Ar), 5.13 (s, 1H, CH), 3.87 (d, *J* = 7.55 Hz, 2H, CH_2_). ^13^CNMR (125 MHz, DMSO-*d*_*6*_): *δ* 162.5, 165.2, 158.5, 157.7, 152.1, 150.2, 149.2, 147.2, 136.6, 124.3, 120.0, 114.7, 113.2, 110.1, 107.4, 104.5, 64.5, 55.4, 33.4. HREI-MS: m/z calcd for C_19_H_14_FN_3_O_7_S [M]^+^ 447.0536, Found: 447.0524.

#### 4-Fluoro-6-nitro-N-(4-(2-nitrophenyl)-2-oxothiazolidin-3-yl)benzofuran-2-carboxamide (3)

Yield: 78%; ^1^HNMR (500 MHz, DMSO-*d*_6_): *δ* 12.69 (s, 1H, NH), 8.95 (s, 1-H, Ar), 8.39 (d, *J* = 7.55 Hz, 1H, Ar), 8.14 (dd, *J* = 6.50, 6.85 Hz, 1H, Ar), 7.97 (d, *J* = 8.35 Hz, 2H, Ar), 7.86 (t, *J* = 6.25 Hz, 1-H, Ar), 7.73 (t, *J* = 6.45 Hz, 1H, Ar), 5.12 (s, 1-H, CH), 3.86 (d, *J* = 7.57 Hz, 2H, CH_2_). ^13^CNMR (125 MHz, DMSO-*d*_*6*_): *δ* 165.2, 162.5, 158.5, 157.7, 152.1, 150.2, 149.1, 138.4, 135.8, 128.9, 128.2, 125.6, 124.3, 110.1, 107.4, 104.5, 64.5, 33.4. HREI-MS: m/z calcd for C_18_H_11_FN_4_O_7_S [M]^+^ 446.0332, Found 446.0319.

#### N-(4-(4-(benzyloxy)phenyl)-2-oxothiazolidin-3-yl)-4-fluoro-6-nitrobenzofuran-2-carboxamide (4)

Yield: 77%; ^1^H-NMR (500 MHz, DMSO-*d*_6_): *δ* 12.22 (s, 1H, NH), 8.84 (d, *J* = 1.8 Hz, 1H, Ar), 8.37 (q, 1H, Ar), 7.97 (d, *J* = 7.6 Hz, 1H, Ar), 7.92 (s, 1H, Ar), 7.71 (d, *J* = 7.2 Hz, 1H, Ar), 7.48 (d, *J* = 6.1 Hz, 1H, Ar), 7.42 (t, *J* = 6.1 Hz, 2H, Ar), 7.36 (d, *J* = 6.05 Hz, 1H, Ar), 7.13 (d, *J* = 7.25 Hz, 2H, Ar), 5.17 (s, 1H, CH), 3.88 (d, *J* = 7.53 Hz, 2H, CH_2_). ^13^CNMR (125 MHz, DMSO-*d*_*6*_): *δ* 165.2, 162.5, 158.5, 157.7, 157.9, 152.1, 150.2, 137.3, 136.1, 129.2, 129.2, 128.9, 128.2, 128.2, 127.4, 127.4, 124.3, 115.4, 115.4, 110.1, 107.4, 104.5, 71.4, 64.5, 33.4. HREI-MS: m/z calcd for C_25_H_18_FN_3_O_6_S [M]^+^ 507.0900, Found 507.0887.

#### 4-Fluoro-N-(4-(4-hydroxyphenyl)-2-oxothiazolidin-3-yl)-6-nitrobenzofuran-2-carboxamide (5)

Yield: 88%; ^1^H-NMR (500 MHz, DMSO-*d*_6_): *δ* 12.12 (s, 1H, NH), 8.84 (d, *J* = 1.85 Hz, 1H, Ar), 8.41 (s, 1H, Ar), 8.37 (dd, *J* = 2 Hz, 1H, Ar), 7.96 (d, *J* = 7.6 Hz, 1H, Ar), 7.90 (s, 1H, Ar), 7.60 (d, *J* = 7.1 Hz, 2H, Ar), 6.86 (d, *J* = 7.1 Hz, 1H, Ar), 3.87 (d, *J* = 7.50 Hz, 2H, CH_2_). ^13^CNMR (125 MHz, DMSO-*d*_*6*_): *δ* 165.2, 162.5, 158.5, 157.1, 157.9, 152.1, 150.2, 138.4, 129.1, 129.1, 124.3, 117.3, 117.3, 110.1, 107.4, 104.5, 64.5, 33.4. HREI-MS: m/z calcd for C_18_H_12_FN_3_O_6_S [M]^+^ 417.0430, Found 417.0413.

#### 4-Fluoro-6-nitro-N-(4-(4-nitrophenyl)-2-oxothiazolidin-3-yl)benzofuran-2-carboxamide (6)

Yield: 70%; ^1^HNMR (500 MHz, DMSO-*d*_6_): *δ* 10.79 (s, 1-H, NH), 8.82 (d, *J* = 1.85 Hz, 1-H, Ar), 8.36 (q, *J* = 1.85 Hz, 2-H, Ar), 7.95 (d, *J* = 7.6 Hz, 2H, Ar), 7.87 (s, 1H, Ar), 5.12 (d, *J* = 7.1 Hz, 1H, Ar), 3.87 (d, *J* = 7.58 Hz, 2H, CH_2_). ^13^CNMR (125 MHz, DMSO-*d*_*6*_): *δ* 165.2, 162.5, 158.5, 157.1, 157.9, 152.1, 150.2, 147.4, 125.3, 125.3, 124.9, 124.9, 124.3, 110.1, 107.4, 104.5, 64.5, 33.4. HREI-MS: m/z calcd for C_18_H_11_FN_4_O_7_S [M]^+^ 446.0332, Found 446.0314.

#### 4-Fluoro-N-(4-(4-hydroxy-3-methoxyphenyl)-2-oxothiazolidin-3-yl)-6-nitrobenzofuran-2-carboxamide (7)

Yield: 76%; ^1^HNMR (500 MHz, DMSO-*d*_6_): *δ* 10.79 (s, NH, 1H), 8.82 (d, *J* = 2 Hz, 2-H, Ar), 8.36 (q, *J* = 2.05 Hz, 2-H, Ar), 7.95 (d, *J* = 7.65 Hz, 2H, Ar), 7.87 (s, 1H, Ar), 5.12 (d, *J* = 7.1 Hz, 1H, Ar), 3.98 (s, 3H, OCH_3_), 3.87 (d, *J* = 7.48 Hz, 2H, CH_2_). ^13^CNMR (125 MHz, DMSO-*d*_*6*_): *δ* 165.2, 162.5, 158.5, 157.1, 157.9, 152.1, 150.2, 147.9, 147.5, 138.7, 124.3, 120.5, 117.3, 111.6, 110.1, 107.4, 104.5, 64.5, 33.4. HREI-MS: m/z calcd for C_19_H_14_FN_3_O_7_S [M]^+^ 447.0536, Found: 447.0524.

#### N-(4-(2,4-dichlorophenyl)-2-oxothiazolidin-3-yl)-4-fluoro-6-nitrobenzofuran-2-carboxamide (8)

Yield: 73%; ^1^H-NMR (500 MHz, DMSO-*d*_6_): *δ* 12.5 (s, 1H, NH), 8.88 (s, 2H, Ar), 8.86 (d, *J* = 1.75 Hz, 2H, Ar), 8.38 (dd, *J* = 1.95 Hz, 2H, Ar), 8.05 (d, *J* = 7.05 Hz, 1H, Ar), 7.97 (d, *J* = 6.4 Hz, 2H, Ar), 7.75 (d, *J* = 1.55 Hz, 1H, Ar), 7.55 (dd, *J* = 1.6, 1 Hz, 1H, Ar), 5.12 (d, *J* = 7.1 Hz, 1H, Ar), 3.87 (d, *J* = 7.57 Hz, 2H, CH_2_). ^13^CNMR (125 MHz, DMSO-*d*_*6*_): *δ* 165.2, 162.5, 158.5, 157.1, 153.2, 151.3, 150.2, 143.4, 135.1, 132.6, 127.8, 124.3, 120.4, 110.1, 107.4, 104.5, 64.5, 33.4. HREI-MS: m/z calcd for C_18_H_10_FN_3_O_5_S [M]^+^ 468.9702, Found: 468.9689.

#### N-(4-(1,3-diphenylpropyl)-2-oxothiazolidin-3-yl)-4-fluoro-6-nitrobenzofuran-2-carboxamide (9)

Yield: 79%; ^1^H-NMR (500 MHz, DMSO-*d*_6_): *δ* 11.18 (s, 1H, NH), 8.85 (d, *J* = 1.75 Hz, 3H, Ar), 8.38 (q, *J* = 1.95 Hz, 4H, Ar), 7.99 (d, *J* = 7.55 Hz, 3H, Ar), 7.93 (s, 3H, Ar), 5.12 (d, *J* = 7.1 Hz, 1H, Ar), 3.87 (d, *J* = 7.46 Hz, 2H, CH_2_), 1.2 (d, *J* = 3.1 Hz, 5H, aliphatic). ^13^CNMR (125 MHz, DMSO-*d*_*6*_): *δ* 165.2, 162.5, 158.5, 157.1, 151.3, 150.2, 144.1, 138.4, 131.7, 131.7, 129.9, 129.9, 129.6, 129.6, 128.8, 128.8, 127.6, 126.4, 124.3, 110.1, 107.4, 104.5, 64.5, 41.5, 34.6, 33.9, 33.4. HREI-MS: m/z calcd for C_27_H_22_FN_3_O_5_S [M]^+^ 519.1264, Found: 519.1252.

#### 4-Fluoro-N-(4-(naphthalen-2-yl)-2-oxothiazolidin-3-yl)-6-nitrobenzofuran-2-carboxamide (10)

Yield: 74%; ^1^HNMR (500 MHz, DMSO-*d*_6_): *δ* 10.02 (s, 1H, NH), 8.87 (d, *J* = 1.6 Hz, 1H, Ar), 8.82 (d, *J* = 1.96 Hz, 1H, Ar), 8.69 (s, 1H, Ar), 8.39 (dd, *J* = 1.9, 7.55 Hz, 1H, Ar), 8.20 (s, 1H, Ar), 8.06 (m, 3H, Ar), 7.60 (m, 1H, Ar), 5.12 (d, *J* = 7.1 Hz, 1H, Ar), 3.47 (t, *J* = 1.8 Hz, 2H, CH_2_). ^13^CNMR (125 MHz, DMSO-*d*_6_): *δ* 165.2, 162.5, 158.5, 157.1, 151.3, 150.2, 136.3, 134.7, 132.3, 129.4 128.9, 128.4, 128.0, 127.6, 126.8, 125.1, 124.3, 110.1, 107.4, 104.5, 64.5, 33.4. HREI-MS: m/z calcd for C_22_H_14_FN_3_O_5_S [M]^+^ 451.0638, Found: 451.0619.

#### N-(4-(3,5-dichloro-2-hydroxyphenyl)-2-oxothiazolidin-3-yl)-4-fluoro-6-nitrobenzofuran-2-carboxamide (11)

Yield: 75%; ^1^HNMR (500 MHz, DMSO-*d*_6_): *δ* 12.16 (s, 1H, NH), 8.84 (s, 1H, OH), 8.64 (s, 1H, Ar), 8.37 (d, *J* = 7.35 Hz, 1H, Ar), 7.97 (d, *J* = 12 Hz, 1H, Ar), 7.67 (s, 1H, Ar), 5.12 (d, *J* = 7.1 Hz, 1H, Ar), 3.47 (t, *J* = 1.8 Hz, 2H, CH_2_). ^13^CNMR (125 MHz, DMSO-*d*_*6*_): *δ* 165.2, 162.5, 158.5, 157.1, 151.3, 150.2, 147.8, 135.1, 130.7, 129.4, 127.5, 124.3, 120.0, 110.1, 107.4, 104.5, 64.5, 33.4. HREI-MS: m/z calcd for C_18_H_10_Cl_2_FN_3_O_6_S [M]^+^ 484.9651, Found 484.9639.

#### 4-Fluoro-N-(4-(2-hydroxynaphthalen-1-yl)-2-oxothiazolidin-3-yl)-6-nitrobenzofuran-2-carboxamide (12)

Yield: 77%; ^1^H-NMR (500 MHz, DMSO-*d*_6_): *δ* 12.99 (s, 1H, OH), 12.51 (s, 1H, NH), 9.56 (t, *J* = 10.1 Hz, 1H, Ar), 8.86 (q, *J* = 9.25 Hz, 1H, Ar), 1H, Ar), 8.39 (dd, *J* = 7.4 Hz, 2H, Ar), 8.0 (m, 4H, Ar), 7.65 (t, *J* = 6.15 Hz, 1H, Ar), 7.44 (t, *J* = 6.2 Hz, 1H, Ar), 7.25 (d, *J* = 7.35 Hz, 1H, Ar), 5.12 (d, *J* = 7.1 Hz, 1H, Ar), 3.47 (t, *J* = 1.9 Hz, 2H, CH_2_). ^13^CNMR (125 MHz, DMSO-*d*_*6*_): *δ* 165.2, 162.5, 158.5, 157.1, 154.6, 151.3, 150.2, 135.4, 129.7, 129.1, 128.8, 127.7, 125.8, 125.5, 124.3, 120.0, 116.3, 110.1, 107.4, 104.5, 64.5, 33.4. HREI-MS: m/z calcd for C_22_H_14_FN_3_O_6_S [M]^+^ 467.0587, Found 467.0578.

#### N-(4-(2,4-dimethylphenyl)-2-oxothiazolidin-3-yl)-4-fluoro-6-nitrobenzofuran-2-carboxamide (13)

Yield: 74%; ^1^H-NMR (500 MHz, DMSO-*d*_6_): *δ* 12.23 (s, 1H, NH), 8.84 (d, *J* = 1.85 Hz, 1H, Ar), 8.79 (s, 1H, Ar), 8.38 (q, *J* = 1.95 Hz, 1H, Ar), 7.97 (t, *J* = 7.55 Hz, 2H, Ar), 7.77 (d, *J* = 6.4 Hz, 1H, Ar), 5.12 (d, *J* = 6.2 Hz, 1H, Ar), 3.47 (t, *J* = 3.1 Hz, 2H, CH_2_), 2.41 (s, 3H, CH_3_), 2.31 (s, 3H, CH_3_). ^13^CNMR (125 MHz, DMSO-*d*_*6*_): *δ* 165.2, 162.5, 158.5, 157.1, 151.3, 150.2, 139.7, 137.5, 136.1, 131.7, 127.6, 126.5, 124.3, 110.1, 107.4, 104.5, 64.5, 33.4, 22.4, 21.7. HREI-MS: m/z calcd for C_20_H_16_FN_3_O_5_S [M]^+^ 429.0794, Found: 429.0782.

#### 4-Fluoro-6-nitro-N-(4-(3-nitrophenyl)-2-oxothiazolidin-3-yl)benzofuran-2-carboxamide (14)

Yield: 72%; ^1^H-NMR (500 MHz, DMSO-*d*_6_): *δ* 12.56 (s, 1H, NH), 8.84 (s, 1H, Ar), 8.61 (s, 1H, Ar), 8.37 (m, 2H, Ar), 8.06 (m, 3H, Ar), 5.12 (d, *J* = 6.2 Hz, 1H, Ar), 3.47 (t, *J* = 3.1 Hz, 2H, CH_2_). ^13^CNMR (125 MHz, DMSO-*d*_*6*_): *δ* 165.2, 162.5, 158.5, 157.1, 151.3, 150.2, 148.9, 145.4, 135.3, 130.1, 124.3, 122.5, 121.8, 110.1, 107.4, 104.5, 64.5, 33.4, HREI-MS: m/z calcd for C_18_H_11_FN_4_O_7_S [M]^+^ 446.0332, Found: 446.0314.

### Urease assay protocol

25 µL of enzyme and 55 µL of buffer was added to 100 mM urea that were incubated with test scaffolds 5 µL (0.5 mM concentration) for 15 min at 30 °C in 96-well plates. Urea concentrations were changed from 2–24 mM to study kinetics. As described by Weatherburn the urease potential was assessed by measuring the ammonia production with using indophenol method^35^. Concisely, 70 µL of alkali reagent (0.1% active chloride NaOCl and 0.5 w/v NaOH) and 45 µL of reagent phenol (0.005% w/v sodium nitroprusside and 1% w/v phenol) were added to each and every well plate. Using microplate reader (Molecular Device, USA) the increase in absorbance at 630 nm was measured after 50 min. Using 200 µL as final volume the reading was performed as triplicate. With help of SoftMaxPro software (Molecular Device, USA) the results (per min absorbance change) were calculated. At pH 6.8 the entire assay was performed. With the help of 100 − (OD_test well_/OD_control_) × 100 the percentage inhibition was calculated. For urease inhibition thiourea was used as standard.

## Supplementary information

Supplementary information
