# Supplementary material for: Synthesis, in vitro urease inhibitory potential and molecular docking study of benzofuran-based-thiazoldinone analogues
Source: Sci Rep. 2020 Jun 30;10:10673. doi: 10.1038/s41598-020-67414-7 (PMC7326984; doi:10.1038/s41598-020-67414-7)
Supplement: Supplementary file 1 — Supplementary information [file 41598_2020_67414_MOESM1_ESM.docx]

**Synthesis, in vitro urease inhibitory potential and molecular docking study of benzofuran-based-thiazoldinone analogues**

**Muhammad Taha**^^[[1]](#footnote-1)^*^**^a^**, **Fazal Rahim^b^**, **Hussan Zada^b^***,* **Manikandan Selvaraj^c^**, **[Rai Khalid Farooq](https://www.sciencedirect.com/science/article/pii/S0045206818301561#!)^d^**, **Syed Adnan Ali Shah ^e,f^**, **Muhammad Nawaz^g^**, **Zainul Amiruddin Zakaria*****^h,i^**

*^a^*Department of clinical pharmacy, Institute for Research and Medical Consultations (IRMC), Imam Abdulrahman Bin Faisal University, P.O. Box 31441, Dammam, Saudi Arabia.

*^b^Department of Chemistry, Hazara University, Mansehra-21300, Khyber Pakhtunkhwa, Pakistan*

^c^Monash University School of chemical engineering, Bandar Subway, 47500 selangor Alam Campus, 42300;

^d^Department of Neuroscience Research, Institute for Research and Medical Consultations (IRMC), Imam Abdulrahman Bin Faisal University, P.O. Box 1982, Dammam 31441, Saudi Arabia

*^e^Atta-ur-Rahman Institute for Natural Product Discovery, Universiti Teknologi MARA (UiTM), Puncak Alam Campus, 42300 Bandar Puncak Alam, Selangor D. E. Malaysia.*

*^f^Faculty of Pharmacy, Universiti Teknologi MARA (UiTM), Puncak Alam Campus, 42300 Bandar Puncak Alam, Selangor Darul Ehsan, Malaysia*

*^g^Department of Nano-Medicine Research, Institute for Research and Medical Consultations (IRMC), Imam Abdulrahman Bin Faisal University, P.O. Box 1982, Dammam 31441, Saudi Arabia*

*^h^Department of Biomedical Science, Faculty of Medicine and Health Sciences, Universiti Putra Malaysia, 43400 Serdang, Selangor, Malaysia*

*^i^Halal Institute Research Institute, Universiti Putra Malaysia, 43400 Serdang, Selangor, Malaysia*


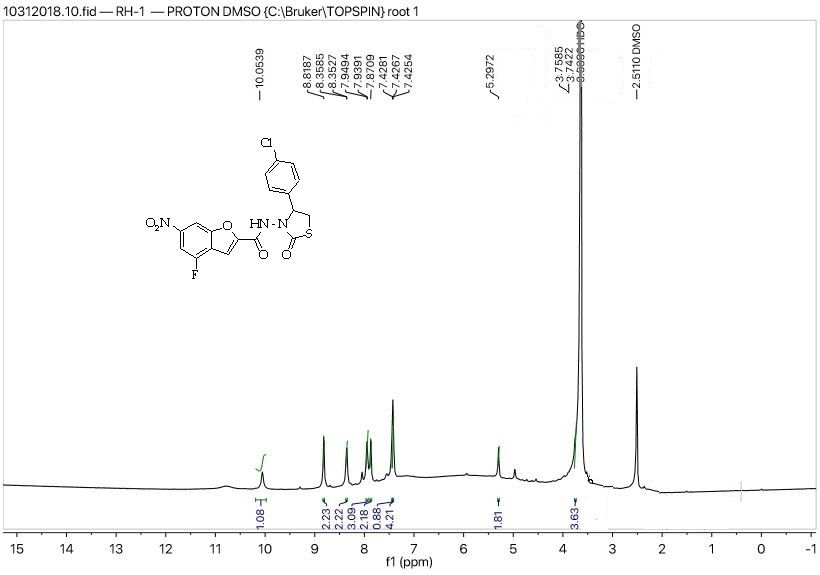


### S-1: ^1^H NMR spectra of *N*-(4-(4-chlorophenyl)-2-oxothiazolidin-3-yl)-4-fluoro-6-nitrobenzofuran-2-carboxamide (1)


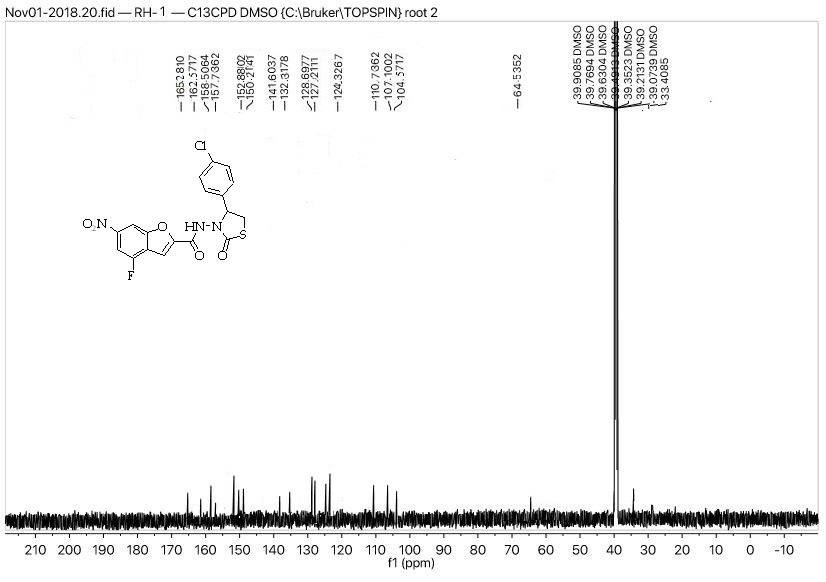


S-2: ^13^C NMR spectra of *N*-(4-(4-chlorophenyl)-2-oxothiazolidin-3-yl)-4-fluoro-6-nitrobenzofuran-2-carboxamide (1)

###
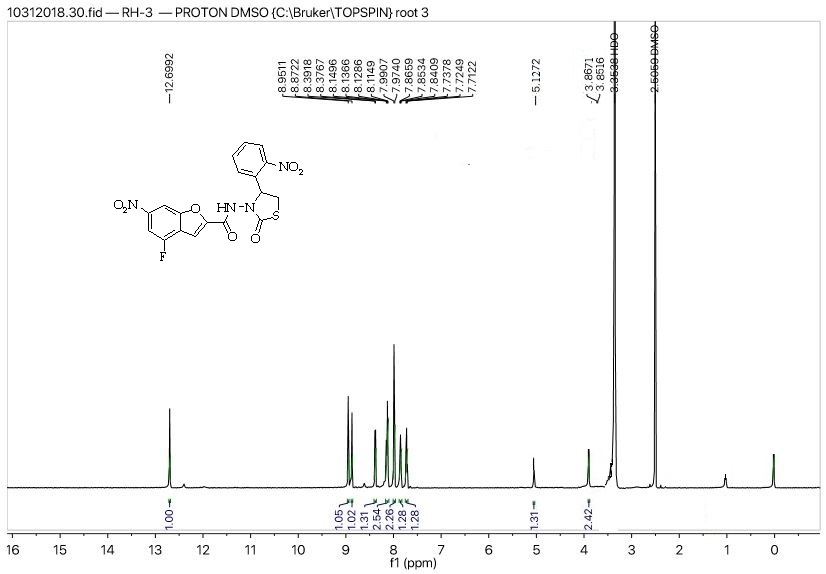


### S-3: ^1^H NMR spectra of 4-fluoro-6-nitro-*N*-(4-(2-nitrophenyl)-2-oxothiazolidin-3-yl)benzofuran-2-carboxamide (3)


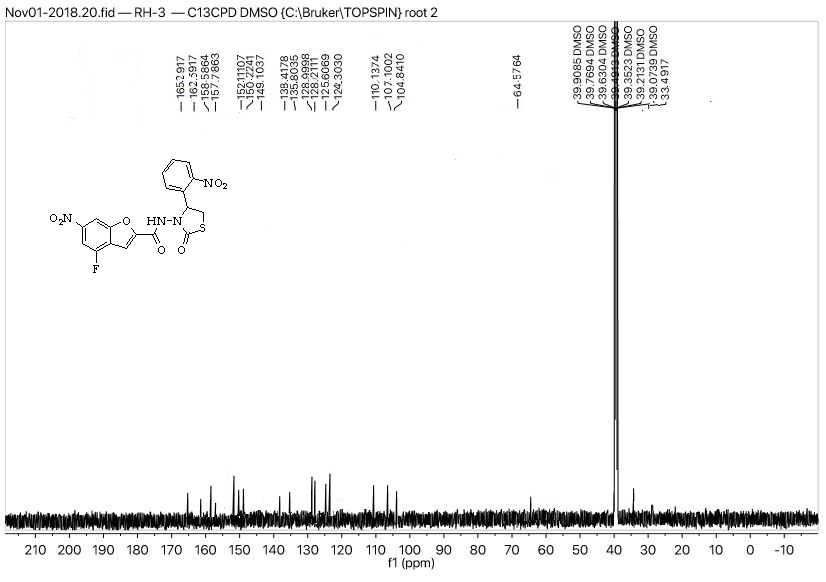
S-4: ^13^C NMR spectra of 4-fluoro-6-nitro-*N*-(4-(2-nitrophenyl)-2-oxothiazolidin-3-yl)benzofuran-2-carboxamide (3)

###
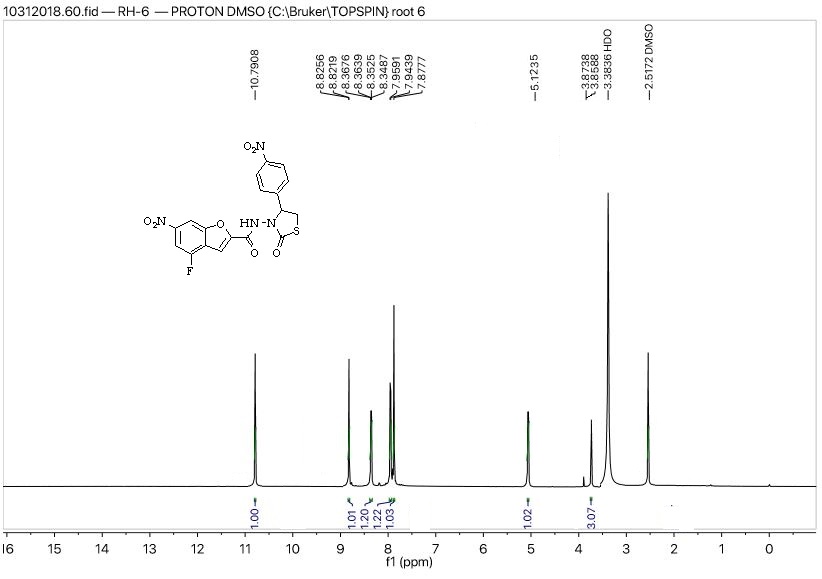


### S-5: ^1^H NMR spectra of 4-fluoro-6-nitro-*N*-(4-(4-nitrophenyl)-2-oxothiazolidin-3-yl)benzofuran-2-carboxamide (6)


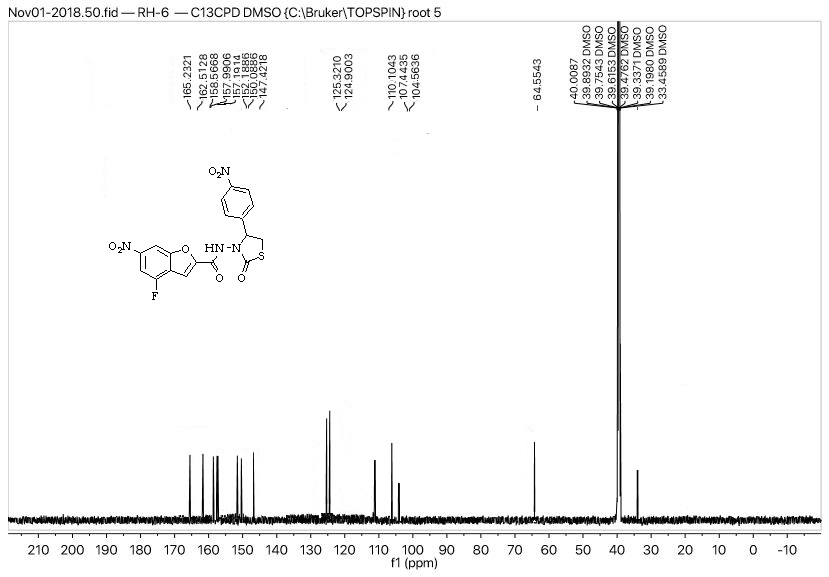


### S-6: ^13^C NMR spectra of 4-fluoro-6-nitro-*N*-(4-(4-nitrophenyl)-2-oxothiazolidin-3-yl)benzofuran-2-carboxamide (6)

1. * Corresponding authors:

   Dr Muhammad taha E-mail: [taha_hej@yahoo.com](mailto:taha_hej@yahoo.com) and mtaha@iau.edu.sa, (MT)

   ^*^Correspondence 2: [zaz@upm.edu.my](mailto:zaz@upm.edu.my) (Tel.: +603-8947 2111; Fax: +603-89436178) [↑](#footnote-ref-1)
